# Towards novel osteoarthritis biomarkers: Multi-criteria evaluation of 46,996 segmented knee MRI data from the Osteoarthritis Initiative

**DOI:** 10.1371/journal.pone.0258855

**Published:** 2021-10-21

**Authors:** Alexander Tack, Felix Ambellan, Stefan Zachow

**Affiliations:** 1 Zuse Institute Berlin, Berlin, Germany; 2 Charité – Universitätsmedizin Berlin, Berlin, Germany; Monash University, AUSTRALIA

## Abstract

Convolutional neural networks (CNNs) are the state-of-the-art for automated assessment of knee osteoarthritis (KOA) from medical image data. However, these methods lack interpretability, mainly focus on image texture, and cannot completely grasp the analyzed anatomies’ shapes. In this study we assess the informative value of quantitative features derived from segmentations in order to assess their potential as an alternative or extension to CNN-based approaches regarding multiple aspects of KOA. Six anatomical structures around the knee (femoral and tibial bones, femoral and tibial cartilages, and both menisci) are segmented in 46,996 MRI scans. Based on these segmentations, quantitative features are computed, i.e., measurements such as cartilage volume, meniscal extrusion and tibial coverage, as well as geometric features based on a statistical shape encoding of the anatomies. The feature quality is assessed by investigating their association to the Kellgren-Lawrence grade (KLG), joint space narrowing (JSN), incident KOA, and total knee replacement (TKR). Using gold standard labels from the Osteoarthritis Initiative database the balanced accuracy (BA), the area under the Receiver Operating Characteristic curve (AUC), and weighted kappa statistics are evaluated. Features based on shape encodings of femur, tibia, and menisci *plus* the performed measurements showed most potential as KOA biomarkers. Differentiation between non-arthritic and severely arthritic knees yielded BAs of up to 99%, 84% were achieved for diagnosis of early KOA. Weighted kappa values of 0.73, 0.72, and 0.78 were achieved for classification of the grade of medial JSN, lateral JSN, and KLG, respectively. The AUC was 0.61 and 0.76 for prediction of incident KOA and TKR within one year, respectively. Quantitative features from automated segmentations provide novel biomarkers for KLG and JSN classification and show potential for incident KOA and TKR prediction. The validity of these features should be further evaluated, especially as extensions of CNN-based approaches. To foster such developments we make all segmentations publicly available together with this publication.

## Introduction

Medical imaging has become the standard diagnostic means for assessing osteoarthritis. Substantial efforts have been made in the past decades to identify image-based biomarkers and to develop methods for image-based assessment of knee osteoarthritis (KOA) from conventional radiographs and tomographic image data. To rate KOA from X-Rays with the knee being in a load-bearing situation, the current gold standard is the Kellgren-Lawrence grading (KLG) [1], where e.g. radiographic joint narrowing (JSN) is measured. To three-dimensionally (3-D) assess an arthritic anatomy, 3-D imaging methods are compulsory [2] (or at least reliable 3-D reconstruction methods from 2-D images [3]). Compared to computed tomography, magnetic resonance imaging (MRI) offers the advantages of no radiation exposure and significantly better differentiation of soft tissues. Various procedures have been proposed for KOA diagnostics from MRI data, such as manual image reading based on semi-quantitative scoring systems [4, 5], computerized quantitative analysis based on manual definitions of regions of interest (ROI) [6–10], up to fully automated methods based on machine learning [11, 12].

In order to gain more insight into the pathogenesis of osteoarthritis and the underlying phenotypes, a big amount of data needs to be studied. Time efficient processing of thousands of MRI scans, however, seems to be feasible only by employing automated methods, which additionally could give rise to a more objective and holistic support of KOA scoring by incorporating multiple data sources. Promising results in view of automated processing were achieved recently using deep learning. However, most of these approaches were designed to perform a single task only, e.g. diagnosis of cartilage degeneration [13, 14] or meniscal lesions [15–17] in MRI data. Methods of deep learning are complex to design, their decision process is hardly explainable, and huge burdens need to be overcome with respect to a generalizability for different imaging modalities [18].

In this study, we evaluate various KOA aspects using different quantitative characteristics of individual structures of the knee in order to investigate their potential as biomarkers. Therefore, we apply an automated segmentation approach for six anatomical structures (femoral and tibial bones, femoral and tibial cartilages, and both menisci) to almost all subjects contained in the Osteoarthritis Initiative (OAI) database (https://nda.nih.gov/oai) and perform thorough quality assurance of our segmentation results. We investigate which features show highest potential for a holistic assessment of KOA by classification of KLG and JSN as well as by predicting a possible occurrence of incident KOA or the need for a total knee replacement (TKR) within a time frame of up to five years. With this work we aim to set a basis for future developments within KOA research and diagnosis by supplementing the OAI database with the segmented structures (https://pubdata.zib.de).

## Materials and methods

### Study population

This study is based on 3D sagittal Double Echo Steady-State (DESS) MRI data acquired by the OAI using Siemens Trio 3.0 Tesla scanners [19]. A total of 48,073 datasets are available (see Table 1), which split up into 7 time points: baseline visit (v00, MRI data of 9,494 knees), 1 year follow-up (v12, 8,187 scans), 2 year follow-up (v24, 7,534 scans), 3 year follow-up (v36, 5,604 scans), 4 year follow-up (v48, 6,743 scans), 6 year follow-up (v72, 5,508 scans), and 8 year follow-up (v96, 5,003 scans).

**Table 1:** Demographics of data used in this study summarized for all considered time points of the OAI database.

Visit	# Images	Side (left, right)	Sex (male, female)	Age [years]	BMI [kg/m^2^]	KLG (0, 1, 2, 3, 4, NA)	mJSN (0, 1, 2, 3, NA)	lJSN (0, 1, 2, 3, NA)
v00	9345	4639, 4706	3847, 5498	61.09 ± 9.18	28.59 ± 4.83	3404, 1565, 2329, 1203, 284, 560	5699, 1909, 978, 199, 560	8089, 361, 248, 87, 560
v12	8025	4006, 4019	3347, 4678	62.13 ± 9.13	28.44 ± 4.79	2954, 1371, 2103, 1130, 328, 139	5054, 1699, 905, 228, 139	7232, 306, 247, 101, 139
v24	7338	3660, 3678	3114, 4224	62.93 ± 9.09	28.39 ± 4.84	2681, 1246, 1914, 1054, 331, 112	4611, 1545, 835, 234, 113	6611, 279, 238, 97, 113
v36	5500	2033, 3467	2356, 3144	63.64 ± 9.06	28.38 ± 4.80	1973, 910, 1429, 835, 260, 93	3414, 1149, 658, 186, 93	4932, 207, 192, 76, 93
v48	6616	3276, 3340	2819, 3797	64.68 ± 9.06	28.45 ± 4.88	2357, 1072, 1690, 924, 332, 241	4094, 1313, 732, 236, 241	5824, 240, 214, 97, 241
v72	5413	2692, 2721	2317, 3096	66.07 ± 8.82	28.25 ± 4.94	1692, 886, 421, 176, 23, 2215	2544, 499, 139, 16, 2215	3034, 113, 43, 8, 2215
v96	4759	2305, 2454	2055, 2704	67.56 ± 8.64	28.38 ± 5.02	1595, 807, 407, 208, 38, 1704	2406, 455, 166, 28, 1704	2887, 115, 44, 10, 1703

NA: ‘Not available’; no measurement was performed within the OAI study for these knees.

All datasets of the retrospective OAI database study are fully anonymized. Ethics approval for the OAI database was obtained by the OAI coordinating center and by each OAI clinical site. All patients provided written informed consent for participation in the OAI and to have their data from their medical records used in research.

### Automatic segmentation of MRI data

**Fig 1:**
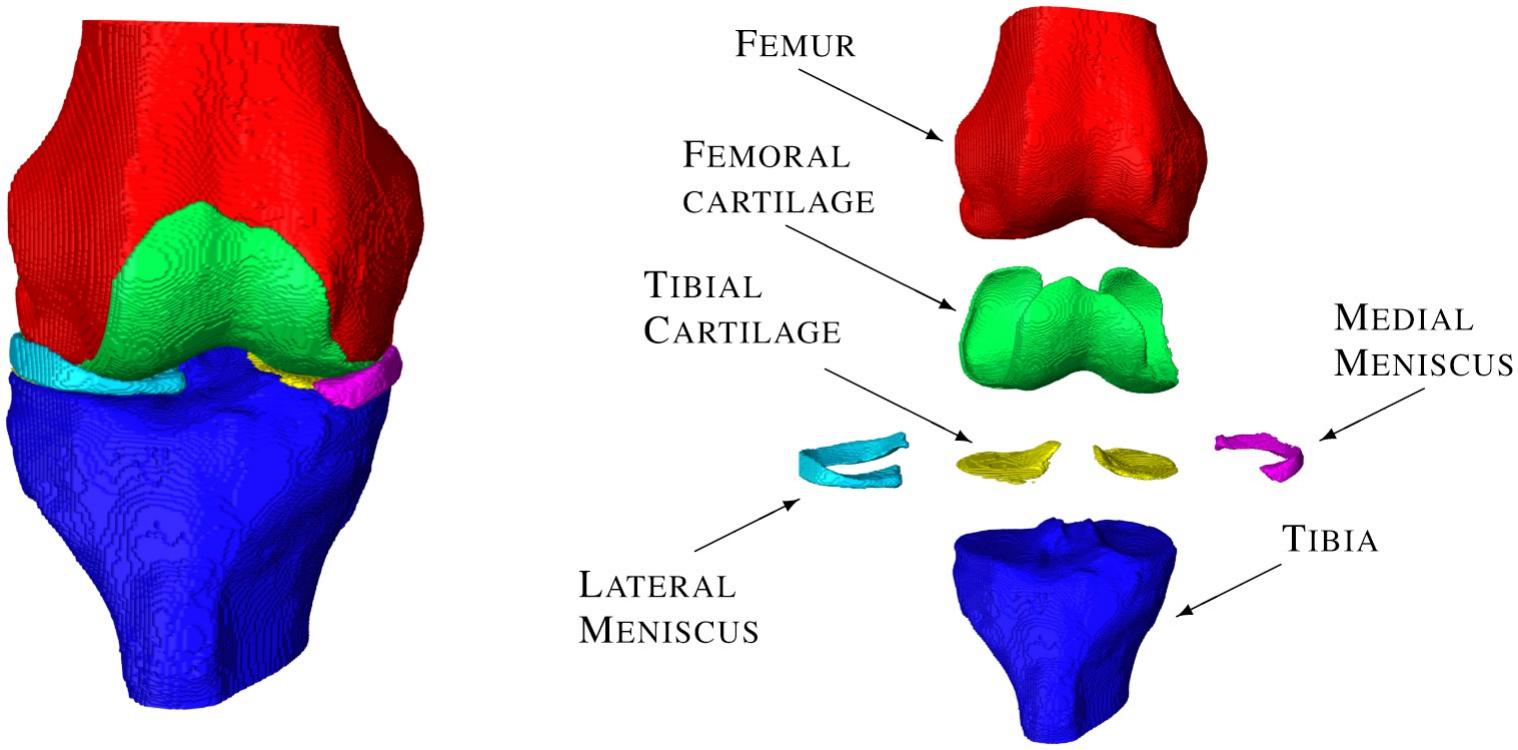
Anatomical regions of interest resulting from the automated segmentation methods. Our automated segmentation methods yield segmentation masks for the femoral bone, tibial bone, femoral and tibial cartilage, and both menisci.

The anatomical structures most affected by KOA are the bones, cartilages and menisci of the knee. To compute features that might be suitable for being used as biomarkers for any or each of these structures individually we first employ methods of image segmentation to specify the anatomical ROIs (see Fig 1). We utilize the method of Ambellan et al. 2019 [20], to segment the distal femoral bone (FB) and the proximal tibial bone (TB) as well as the femoral and tibial cartilage (FC, TC). In addition, the method of Tack et al. 2018 [21], is utilized to segment the medial and lateral meniscus (mM, lM).

Our fully automatic segmentation method has a run time of approx. 10 minutes per MRI dataset on a common workstation. This means the segmentation would almost take a year for all 48,073 datasets on a single machine without any parallelization. To carry out this massive segmentation effort, we employed a combination of high performance computing (HLRN https://www.hlrn.de/supercomputer-e/hlrn-iv-system/?lang=en) and GPU-based application of machine learning (NVIDIA DGX-1 system (NVIDIA Corporation, California, United States), provided by the Max Delbrück Center for Molecular Medicine in Berlin, Germany (https://www.mdc-berlin.de/), which allowed us to segment 48,073 MRI datasets in about 5 weeks.

### Data: Inclusion and exclusion criteria

**Fig 2:**
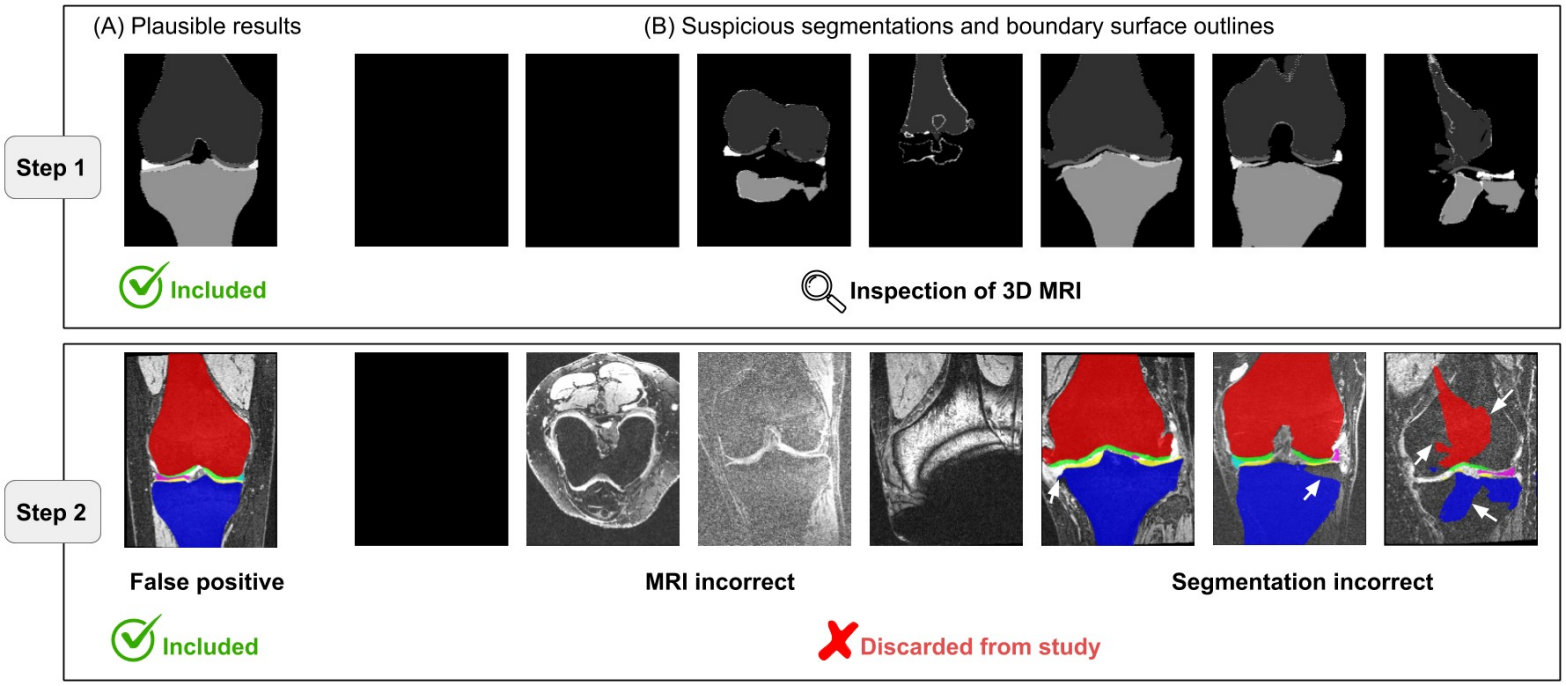
Quality assurance of automated segmentations. Step 1: Segmentation results (outlines) are shown for coronal slices of the MRI. Plausible results (A) are included in this study. For suspicious results (B) the complete 3D MRI is inspected (Step 2). Examples for discarded cases are (from left to right): MRI can not be loaded, wrong MRI orientation, doubtful MRI appearance, MRI artifacts, incorrect menisci segmentation, incorrect bone segmentation, incorrect overall segmentation (incorrect segmentations indicated by white arrows). 1,850 cases which appeared suspicious in Step 1 were identified as false positives in Step 2 and remained included in this study.

The employed methods allowed us to segment several thousand datasets in a fully automated fashion. Our motivation is to include only datasets having a plausible segmentation in the analysis of features to test their possible suitability as KOA biomarkers. To ensure a sufficient quality for such an analysis, we visually inspected each segmented dataset—a process that took roughly 90 person hours. We empirically found coronal slice number 190 (± 5) to typically show all structures of interest since the field of view of the MRI data from the OAI database is well standardized (Fig 2). For an efficient verification of the segmentation results, we first limited ourselves to inspect the aforementioned 11 slices, in which all relevant structures can be seen. Only if there were inconsistencies in the expected segmentation, the whole MRI data set was checked. If any anatomical structure has not been properly segmented or any part of the segmentation looked suspicious in the respective slices, the complete 3D segmentation was again inspected (Fig 2). Out of 48,073 scans 2,927 (6.1%) were selected for additional 3D inspection and 1,077 datasets (2.2%) were excluded from this study due to bad image quality or segmentation failures (S1 Fig). In total 97.8% of all MR images were successfully segmented and included in this study resulting in 46,966 datasets.

### Computation of features

**Fig 3:**
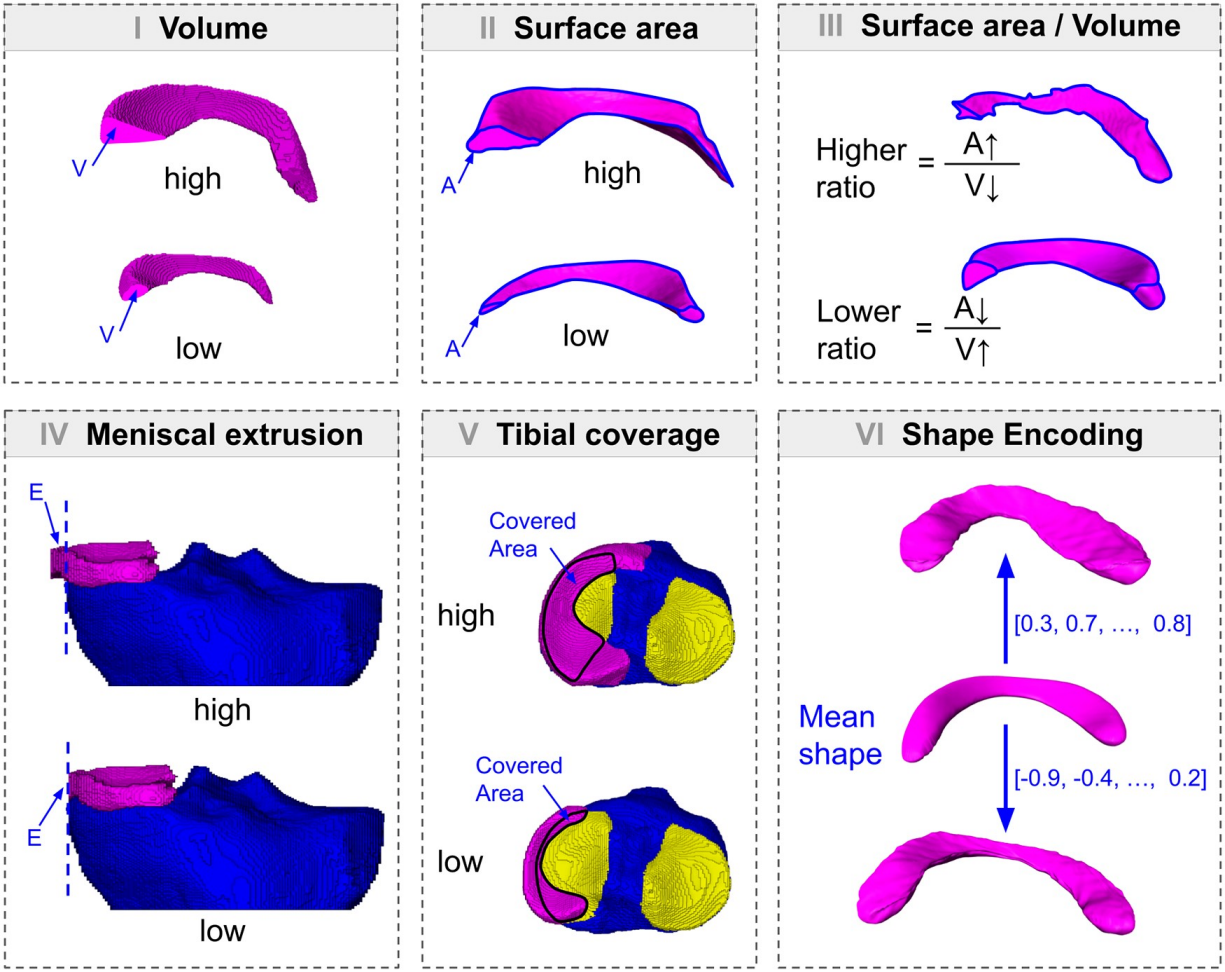
Computation of KOA features. KOA features are exemplarily shown for the medial meniscus (purple), tibial bone (blue) and tibial cartilage (yellow). MEAS features are computed based on segmented voxels and surfaces generated thereof. For MEAS, (I) volumes are computed for femoral cartilage, medial/lateral/total tibial cartilage, and both menisci, (II) surface areas are computed for both menisci, (III) ratio of volume and surface area is computed for both menisci, and (IV) meniscal extrusion is computed for both menisci. (V) Tibial coverage is computed for both tibial plateaus. (VI) LDSE features are computed for femoral bone, tibial bone, and medial/lateral meniscus representing individual shapes relative to the mean shape of the analyzed population.

Our segmentation yields a disjoint set of labeled voxels for all relevant structures that have been identified in a given MR image. To compute features for KOA assessment of anatomical structures, we use different methods to describe their representation. The amount of voxels, for instance, is proportional to both, the MRI resolution as well as the size of the respective structure. Hence, features related to the volume of an anatomical structure can be directly computed from the segmentations. For more sophisticated features describing the shape of a structure the complexity of the data is reduced by considering only the boundaries of the segmented regions. To achieve this, we represent the respective bounding surfaces as triangulated meshes, which well approximate complexly shaped objects [22]. Based on the surface representations, we compute the surface areas and we statistically analyze the variation in shape for a population of surfaces. In our study, we divide all considered features into two groups: (A) Measurements of volumes, areas and distances which are in the following called “MEAS” features, and (B) Features based on a low-dimensional shape encoding “LDSE” (Fig 3). In detail, the following features are investigated:

#### MEAS features

*Volume*. Swelling of knee soft tissues can lead to an increase of volume whereas severe degeneration may lead to a complete loss. Hence, six MEAS features are chosen as the volumes *V* of mM, lM, FC, total TC, medial TC (mTC), and lateral TC (lTC), which are all computed directly from the labeled voxels. Since volumes of anatomical structures of the knee are known to be correlated with the body size of the subjects [23], all volumes are normalized by the subject’s height.

*Surface area and ratio of surface area to volume*. Especially for the menisci the volume may remain unchanged even if the shape changes, i.e., arthritic menisci may become flat while preserving their volume. To analyze this kind of variation, we selected additional four MEAS features as the surface area *A* for mM and lM, as well as the ratio of surface area to volume *R*_*A*,*V*_ for mM and lM, respectively.

*Meniscal extrusion and tibial coverage*. In addition to changes of volume and shape, anatomical structures (such as the menisci) may also show changes of relative positions to other structures within the knee joint. Meniscal extrusion, for example, is highly correlated with tibial coverage, i.e., the more the meniscus is located outside of the joint, the less tibial cartilage is covered [24]. Thus, additional four MEAS features are chosen as the extrusions of mM and lM in medio-lateral direction measured in *mm* as well as coverages of mTC and lTC by the menisci measured as a percentage (see Tack et al. 2018) [21].

#### LDSE features

Arthritic bones commonly develop osteophytes as well as deformations of the articular surfaces. The shape of the menisci might also change in a more complex manner (e.g. local deformations of the surface), which can not be fully captured by the simple surface area and volume features as contained in MEAS. Therefore, in addition to MEAS, we compute geometrical features assessing the shape of distal femurs, proximal tibias, and the menisci based on a statistical LDSE for all 46,996 datasets, which are divided into seven time points of the OAI database. Using methods from Riemannian shape statistics [25, 26], the mean shape of each anatomy is computed for all subjects segmented in every time point. This mean shape is represented by a common parametrization (i.e., a triangulation).

The variation of all training shapes to the mean is analyzed employing Principal Geodesic Analysis [27]. This analysis yields feature vectors that form a compact encoding for every input shape. In our study the length of the feature vector is proportional to the number of subjects analyzed (see S1 Table) and independent of the geometry’s spatial sampling (i.e., the number of sample points of the parametrization).

The feature vectors are ordered by the magnitude of explained variation in shape [28]. We decided to consider only the 300 most significant features per anatomical structure for our analysis since additional features would contribute only with very little variation in shape [29]. The LDSE features in the following investigation are denoted with LDSE-FB for femoral bone, LDSE-TB for tibial bone, LDSE-mM for medial meniscus, and LDSE-lM for lateral meniscus. Additionally, we analyzed combined encodings LDSE-COMB consisting of the first 75 features of each anatomical structure, again resulting in 300 features. An overview of MEAS and LDSE features is shown in S2 Table.

### Suitability of the features as potential KOA biomarkers

**Fig 4:**
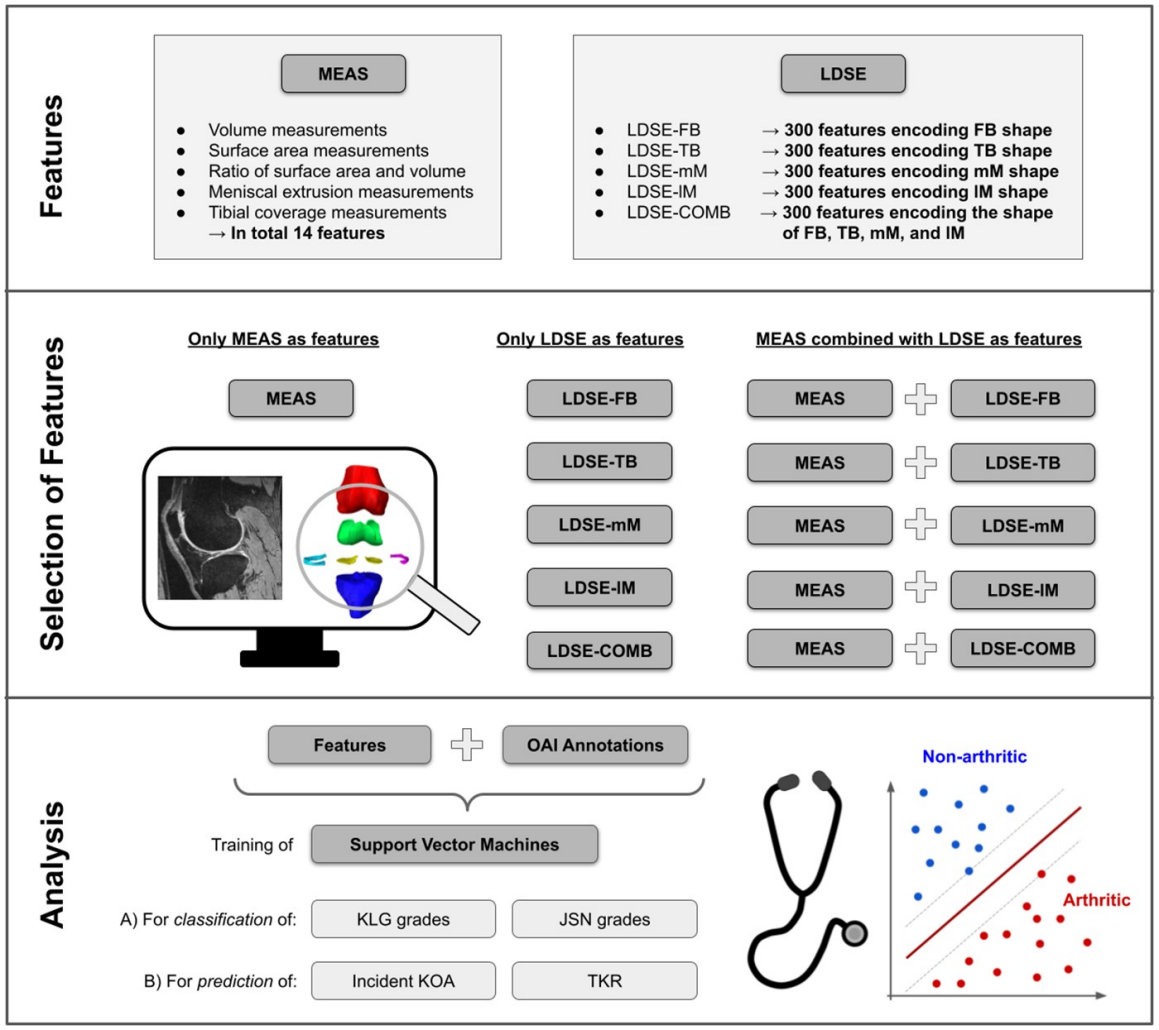
Selection of KOA features and performed analysis. Top: Computation and determination of suitable features. Middle: Selection of features for further analyses. Chosen are either all features of MEAS, or LDSE-FB/LDSE-TB/LDSE-mM/LDSE-lM features encoding *one* anatomy, LDSE-COMB features encoding *all* anatomies, or MEAS combined with one kind of LDSE features. Bottom: Quantitative analysis of the suitability of selected features as KOA biomarkers by training of support vector machines for classification and prediction purposes.

We investigate the potential MEAS and LDSE features (i) to *classify* the current disease state and (ii) to *predict* a disease state which might develop in the future (Table 2). Annotations from image reading studies of the OAI database are used as the gold standard. The entire procedure from the determination of features, their appropriate selection, up to the assessment of their suitability as KOA biomarkers is depicted in Fig 4.

**Table 2:** Analyses performed to investigate the suitability of features to describe different aspects of KOA. Features are employed to classify between KLGs, grades of joint space narrowing (JSN), as well as to predict incident KOA and total knee replacement (TKR).

Classification		Prediction	
KLG	JSN	incident KOA	TKR
0 vs. 1 vs. 2 vs. 3 vs. 4	mJSN: 0 vs. 1 vs. 2 vs. 3	within 1 year	within 1 year
[0;1] vs. 2 vs. [3;4]	lJSN: 0 vs. 1 vs. 2 vs. 3	within 2 years	within 2 years
[0;1] vs. [2;3;4]		within 3 years	within 3 years
0 vs. 2		within 4 years	within 4 years
0 vs. 4		within 5 years	within 5 years

#### Evaluation of features for *classification* purposes

We employed features for *classification* of non-arthritic subjects vs. early KOA and for *classification* of non-arthritic vs. subjects with severe KOA. Non-arthritic subjects were defined as KLG ≤ 1, early KOA as KLG 2, and severe KOA as KLG ≥ 3. In addition, we employed features for the *classification* of medial JSN (mJSN) and lateral JSN (lJSN) (Table 2).

#### Evaluation of features for *prediction* purposes

We evaluated if the analyzed features can *predict* which subjects may develop incident KOA (KLG ≥ 2, with joint space narrowing) as well as which subjects may receive a TKR. Both analyses considered a time frame of up to five years (Table 2).

### Supervised learning via support vector machines

We trained fast and efficient linear support vector machines (SVMs) [30] to classify as well as to predict the aspects of KOA described in the previous section. Standard SVM parameters were chosen (scikit learn: LinearSVC) as well as a squared *L*_2_ penalty [30]. The SVM was utilized in a Monte-Carlo cross-validation framework: For each investigation, data were randomly split into 90% for training and 10% for testing. The data for training and testing were drawn in a balanced fashion limiting all classes to the one with the least subjects. To obtain results which are representative for the complete cohort, this procedure was repeated 1,000 times. Each respective feature *x* was min-max normalized over all training datasets: $z=\frac{x-\text{min}(x)}{x_{\text{max}}-x_{\text{min}}}$.

For each classification setting those cases were discarded that have a missing label (e.g. missing KLG information) or any missing feature.

Balanced accuracy $\text{BA}=\frac{\text{Sensitivity}+\text{Specificity}}{2}$ was used to evaluate the classification of KLG and JSN grades since it can be computed for both, binary and multi-class classification approaches. Additionally, the agreement of our classifications and predictions to the gold standard annotations was assessed employing weighted kappa [31]. The quality of the employed features was evaluated for prediction of incident KOA as well as for prediction of TKR in terms of sensitivity and specificity using the area under the Receiver Operating Characteristic curve (AUC) [32].

### Logistic regression to assess feature importance for prediction of knee arthroplasty

TKR poses an important outcome. For this reason, we perform logistic regression employing MEAS features as independent variables for the prediction of knee arthroplasty. This evaluation is performed for a time frame of one year. The odds ratios (ORs) are provided for each MEAS feature to assess the influence of the respective feature for TKR prediction.

## Results

### Evaluation of features for classification purposes

All results for classification of different grades of KLG (see Table 3) as well as JSN (see Table 4) are averaged over all time points of the OAI study. Detailed information on the results per single time point can be found in the supplementary material.

**Table 3:** Classification of KLG (average over all time points weighted by number of cases).

**Balanced Accuracy**					
	**5-class**	**3-class**	**binary classification**		
Features	0 vs 1 vs 2 vs 3 vs 4	[0;1] vs 2 vs [3;4]	[0;1] vs [2;3;4]	0 vs 2	0 vs 4
	N = 41,932	N = 41,932	N = 41,932	N = 26,949	N = 18,252
MEAS	0.44 ± 0.04	0.60 ± 0.02	0.71 ± 0.02	0.67 ± 0.03	0.94 ± 0.03
LDSE-FB	0.41 ± 0.04	0.64 ± 0.02	0.81 ± 0.02	0.79 ± 0.02	0.95 ± 0.03
LDSE-FB + MEAS	0.45 ± 0.04	0.68 ± 0.02	0.82 ± 0.02	0.80 ± 0.02	0.97 ± 0.02
LDSE-TB	0.40 ± 0.04	0.62 ± 0.03	0.79 ± 0.02	0.77 ± 0.02	0.92 ± 0.04
LDSE-TB + MEAS	0.43 ± 0.04	0.67 ± 0.02	0.80 ± 0.02	0.78 ± 0.02	0.96 ± 0.03
LDSE-mM	0.42 ± 0.04	0.63 ± 0.02	0.77 ± 0.02	0.75 ± 0.02	0.91 ± 0.04
LDSE-mM + MEAS	0.47 ± 0.04	0.67 ± 0.02	0.78 ± 0.02	0.76 ± 0.02	0.97 ± 0.02
LDSE-lM	0.37 ± 0.04	0.56 ± 0.02	0.72 ± 0.02	0.69 ± 0.02	0.88 ± 0.05
LDSE-lM + MEAS	0.44 ± 0.04	0.64 ± 0.02	0.76 ± 0.02	0.73 ± 0.03	0.96 ± 0.03
LDSE-COMB	0.50 ± 0.04	0.72 ± 0.02	0.84 ± 0.01	0.84 ± 0.02	0.99 ± 0.02
LDSE-COMB + MEAS	0.52 ± 0.04	0.73 ± 0.02	0.84 ± 0.01	0.84 ± 0.02	0.99 ± 0.01
**Weighted Kappa**					
	**5-class**	**3-class**	**binary classification**		
Features	0 vs 1 vs 2 vs 3 vs 4	[0;1] vs 2 vs [3;4]	[0;1] vs [2;3;4]	0 vs 2	0 vs 4
	N = 41,932	N = 41,932	N = 41,932	N = 26,949	N = 18,252
MEAS	0.68 ± 0.05	0.58 ± 0.04	0.41 ± 0.04	0.34 ± 0.05	0.88 ± 0.07
LDSE-FB	0.68 ± 0.05	0.65 ± 0.03	0.62 ± 0.03	0.58 ± 0.04	0.90 ± 0.06
LDSE-FB + MEAS	0.71 ± 0.04	0.70 ± 0.03	0.65 ± 0.03	0.60 ± 0.04	0.94 ± 0.05
LDSE-TB	0.63 ± 0.06	0.61 ± 0.04	0.58 ± 0.03	0.53 ± 0.05	0.85 ± 0.07
LDSE-TB + MEAS	0.68 ± 0.05	0.68 ± 0.04	0.61 ± 0.03	0.56 ± 0.05	0.92 ± 0.05
LDSE-mM	0.64 ± 0.05	0.63 ± 0.04	0.54 ± 0.03	0.50 ± 0.05	0.83 ± 0.08
LDSE-mM + MEAS	0.73 ± 0.05	0.68 ± 0.03	0.56 ± 0.04	0.51 ± 0.04	0.94 ± 0.05
LDSE-lM	0.56 ± 0.06	0.50 ± 0.04	0.44 ± 0.04	0.39 ± 0.05	0.76 ± 0.09
LDSE-lM + MEAS	0.69 ± 0.05	0.64 ± 0.03	0.52 ± 0.03	0.45 ± 0.05	0.91 ± 0.06
LDSE-COMB	0.78 ± 0.04	0.75 ± 0.03	0.68 ± 0.03	0.68 ± 0.04	0.97 ± 0.03
LDSE-COMB + MEAS	0.78 ± 0.04	0.75 ± 0.03	0.69 ± 0.03	0.68 ± 0.04	0.98 ± 0.03

**Table 4:** Balanced accuracy and weighted kappa for classification of joint space narrowing (JSN) for medial and lateral femorotibial compartment averaged over all time points weighted by number of cases per time point.

Balanced Accuracy			Weighted Kappa		
	Medial	Lateral		Medial	Lateral
Features	N = 41,932	N = 41,932	Features	N = 41,932	N = 41,932
MEAS	0.54 ± 0.07	0.57 ± 0.11	MEAS	0.72 ± 0.08	0.72 ± 0.14
LDSE-FB	0.43 ± 0.08	0.40 ± 0.11	LDSE-FB	0.50 ± 0.13	0.42 ± 0.20
LDSE-FB + MEAS	0.48 ± 0.08	0.46 ± 0.12	LDSE-FB + MEAS	0.60 ± 0.12	0.57 ± 0.18
LDSE-TB	0.44 ± 0.08	0.40 ± 0.12	LDSE-TB	0.50 ± 0.13	0.42 ± 0.22
LDSE-TB + MEAS	0.49 ± 0.08	0.46 ± 0.12	LDSE-TB + MEAS	0.61 ± 0.12	0.58 ± 0.17
LDSE-mM	0.52 ± 0.08	0.33 ± 0.11	LDSE-mM	0.67 ± 0.11	0.24 ± 0.24
LDSE-mM + MEAS	0.56 ± 0.08	0.46 ± 0.12	LDSE-mM + MEAS	0.72 ± 0.08	0.53 ± 0.20
LDSE-lM	0.40 ± 0.08	0.49 ± 0.10	LDSE-lM	0.42 ± 0.14	0.57 ± 0.14
LDSE-lM + MEAS	0.48 ± 0.08	0.51 ± 0.11	LDSE-lM + MEAS	0.62 ± 0.12	0.59 ± 0.15
LDSE-COMB	0.56 ± 0.08	0.50 ± 0.12	LDSE-COMB	0.71 ± 0.10	0.64 ± 0.16
LDSE-COMB + MEAS	0.57 ± 0.09	0.52 ± 0.12	LDSE-COMB + MEAS	0.73 ± 0.09	0.67 ± 0.15

#### Classification of Kellgren-Lawrence grades

We conducted 4 classification studies in which different grades of KLG were differentiated.

1. Non-arthritic knees were classified against severely arthritic ones, i.e., KLG 0 vs. KLG 4. The resulting BA ranged from 88% ± 5% using LDSE-lM features up to 99% ± 1% using LDSE-COMB features *plus* MEAS features.
2. Since KLG 2 is regarded as the first grade at which safe indicators are visible to reliably confirm KOA, whereas KLG 0 (no abnormalities) is without any doubt considered as a non-arthritic knee, non-arthritic knees were classified against early KOA, i.e., KLG 0 vs. KLG 2. Here, MEAS yielded the lowest BA (67% ± 3%). LDSE-COMB features *plus* MEAS features performed best, resulting in a BA of 84% ± 2%.
3. The classification of non-arthritic knees (KLG [0;1]) against diseased ones (KLG [2;3;4]) confirmed our previous finding: MEAS features yielded the lowest BA (71% ± 2%) and LDSE-COMB features *plus* MEAS features performed best resulting in a BA of 84% ± 1%. For classification of KLG [0;1] vs. KLG 2 vs. KLG [3;4] in a 3-class setting the BA slightly decreased to 60% ± 2% for MEAS features and 73% ± 2% for LDSE-COMB features *plus* MEAS features.
4. Differentiation between all 5 grades of KOA is the most challenging task. The weakest differentiation between these five grades was possible using LDSE-lM features (37% ± 4%). Best results were achieved using LDSE-COMB (50% ± 4%) which was further improved using LDSE-COMB features *plus* MEAS features (52% ± 4%).

In all classification studies, LDSE-FB features performed better than LDSE-TB features in terms of BA with a margin of at least 1 and up to 3 percent. Also, the LDSE-mM features always performed better than the LDSE-lM features with a margin of at least 3 and up to 7 percent. LDSE-FB features usually scored better or at least similar good as LDSE-mM features. MEAS features never scored better than the LDSE-COMB features, however, a combination of both features mostly improved the classification results in the 3-class and the 5-class setting. Employing LDSE-COMB features *plus* MEAS features, a moderate agreement was found as measured with the weighted kappa coefficient of 0.75 ± 0.03 and 0.78 ± 0.04 for the 3-class and the 5-class analysis, respectively.

#### Classification of joint space narrowing

We investigated the potential of the features for differentiation of different grades of mJSN and lJSN, respectively.

1. For a classification of mJSN the LDSE-lM features yielded the lowest accuracy (BA of 40% ± 8%). Utilizing LDSE-FB resulted in similar results as LDSE-TB (BA of 43% ± 8% and 44% ± 8%). Among the LDSE features, LDSE-mM yielded best results (BA of 52% ± 8%), however, not as good as LDSE-COMB (BA of 56% ± 8%). MEAS features scored slightly lower than LDSE-COMB (BA of 54% ± 7%). LDSE-COMB *plus* MEAS led to the best results, i.e., a BA of 57% ± 9%.
2. For a classification of lJSN the LDSE-mM features yielded the lowest accuracy (BA of 33% ± 11%). LDSE-FB performed comparable to LDSE-TB (BA of 40% ± 11% and 40% ± 12%, respectively). Among the individual LDSE features, LDSE-lM yielded best results (BA of 49% ± 10%), which was close to LDSE-COMB (BA of 50% ± 12%). The combination with MEAS features improved the results for all individual LDSE features as well as for LDSE-COMB. However, MEAS features alone yielded the best results for classification of lJSN (57% ± 11%).

In terms of kappa statistics the best results were achieved using LDSE-COMB *plus* MEAS for mJSN and MEAS for lJSN yielding moderate agreement in both cases (average weighted kappa of 0.73 ± 0.09 and 0.72 ± 0.14, respectively).

### Evaluation of features for prediction purposes

Results for a prediction of incident KOA and TKR within different periods of time (see Table 5) are summarized in this section.

**Table 5:** Prediction of incident KOA (KLG >= 2 with JSN) and TKR surgery within *a* years. In both investigations the AUC is evaluated utilizing the respective true positive rate as well as the false positive rate.

**Incident KOA—AUC**					
	*a* = 1 year	*a* = 2 years	*a* = 3 years	*a* = 4 years	*a* = 5 years
	#No_INC = 26,302	#No_INC = 25,890	#No_INC = 25,647	#No_INC = 25,395	#No_INC = 25,225
Features	#INC = 372	#INC = 784	#INC = 1,027	#INC = 1,279	#INC = 1,449
MEAS	0.60 ± 0.11	0.60 ± 0.09	0.59 ± 0.08	0.59 ± 0.07	0.59 ± 0.07
LDSE-FB	0.55 ± 0.12	0.56 ± 0.09	0.56 ± 0.08	0.56 ± 0.07	0.56 ± 0.07
LDSE-FB + MEAS	0.55 ± 0.12	0.57 ± 0.09	0.57 ± 0.08	0.57 ± 0.07	0.57 ± 0.07
LDSE-TB	0.54 ± 0.11	0.52 ± 0.10	0.53 ± 0.08	0.54 ± 0.07	0.53 ± 0.07
LDSE-TB + MEAS	0.56 ± 0.12	0.55 ± 0.10	0.54 ± 0.08	0.55 ± 0.07	0.55 ± 0.07
LDSE-mM	0.55 ± 0.11	0.56 ± 0.09	0.56 ± 0.08	0.56 ± 0.07	0.56 ± 0.07
LDSE-mM + MEAS	0.57 ± 0.11	0.56 ± 0.09	0.56 ± 0.09	0.56 ± 0.07	0.57 ± 0.07
LDSE-lM	0.54 ± 0.10	0.55 ± 0.09	0.55 ± 0.08	0.55 ± 0.07	0.55 ± 0.07
LDSE-lM + MEAS	0.55 ± 0.11	0.56 ± 0.09	0.56 ± 0.09	0.57 ± 0.08	0.57 ± 0.07
LDSE-COMB	0.59 ± 0.11	0.59 ± 0.09	0.59 ± 0.08	0.59 ± 0.07	0.59 ± 0.07
LDSE-COMB + MEAS	0.61 ± 0.12	0.59 ± 0.09	0.60 ± 0.08	0.59 ± 0.07	0.59 ± 0.07
**TKR—AUC**					
	*a* = 1 year	*a* = 2 years	*a* = 3 years	*a* = 4 years	*a* = 5 years
	#No_TKR = 46,575	#No_TKR = 46,281	#No_TKR = 45,992	#No_TKR = 45,726	#No_TKR = 45,481
Features	#TKR = 421	#TKR = 715	#TKR = 1,004	#TKR = 1,270	#TKR = 1,515
MEAS	0.74 ± 0.12	0.73 ± 0.10	0.73 ± 0.08	0.72 ± 0.07	0.72 ± 0.06
LDSE-FB	0.69 ± 0.13	0.71 ± 0.10	0.71 ± 0.08	0.70 ± 0.07	0.70 ± 0.07
LDSE-FB + MEAS	0.72 ± 0.13	0.73 ± 0.09	0.72 ± 0.08	0.72 ± 0.07	0.72 ± 0.06
LDSE-TB	0.65 ± 0.14	0.67 ± 0.10	0.68 ± 0.08	0.67 ± 0.08	0.67 ± 0.07
LDSE-TB + MEAS	0.68 ± 0.13	0.69 ± 0.10	0.70 ± 0.08	0.70 ± 0.07	0.69 ± 0.06
LDSE-mM	0.65 ± 0.14	0.69 ± 0.10	0.69 ± 0.08	0.70 ± 0.08	0.70 ± 0.06
LDSE-mM + MEAS	0.70 ± 0.13	0.72 ± 0.10	0.72 ± 0.08	0.72 ± 0.07	0.73 ± 0.06
LDSE-lM	0.67 ± 0.13	0.68 ± 0.11	0.69 ± 0.09	0.69 ± 0.08	0.69 ± 0.07
LDSE-lM + MEAS	0.70 ± 0.13	0.71 ± 0.10	0.73 ± 0.08	0.73 ± 0.07	0.72 ± 0.06
LDSE-COMB	0.74 ± 0.12	0.74 ± 0.09	0.74 ± 0.08	0.74 ± 0.07	0.73 ± 0.07
LDSE-COMB + MEAS	0.76 ± 0.12	0.75 ± 0.10	0.75 ± 0.08	0.75 ± 0.07	0.74 ± 0.06

#### Prediction of incident knee osteoarthritis

Utilization of individual LDSE features for prediction of incident KOA yielded AUCs in a range from 0.52 to 0.56. Adding MEAS features to the individual LDSE features either led to an increase of the AUC or at least to a similar value, ranging between 0.53 to 0.57.

Both, MEAS features and LDSE-COMB features performed better than the individual features alone. Over all investigated periods of time the prediction of KOA within one year was best. For prediction within one year, the AUC was 0.60 ± 0.11 using LDSE-COMB features *or* MEAS features, and 0.61 ± 0.12 using LDSE-COMB features *plus* MEAS features. However, a prediction of incident KOA within 5 years also led to reasonable results, i.e., an AUC of 0.59 ± 0.07 when utilizing LDSE-COMB features *plus* MEAS features.

Across all considered time periods, all individual LDSE features consistently yielded a lower AUC than the MEAS features and the LDSE-COMB features, respectively. A combination of LDSE-COMB and MEAS features, however, either improved the results or at least yielded to equal results for every period of time that has been analysed.

#### Prediction of total knee replacement

LDSE features led to a prediction of TKR with AUCs ranging from 0.65 to 0.71. MEAS features as well as LDSE-COMB features yielded slightly higher AUC values ranging from 0.72 to 0.74 and from 0.73 to 0.74, respectively. In tendency, the best results were achieved for prediction of TKR within one year. The AUC was 0.74 ± 0.12 using LDSE-COMB features, 0.74 ± 0.12 using MEAS features, and 0.76 ± 0.12 using LDSE-COMB features *plus* MEAS features. However, the prediction of receiving TKR within the next 5 years also led to comparable results, i.e., an AUC of 0.74 ± 0.06 when utilizing LDSE-COMB features *plus* MEAS features.

#### Logistic regression for prediction of total knee replacement

The highest odds ratios were achieved by features related to the MM, such as medial tibial coverage (OR: 7.04, 95% confidence interval (CI): 6.70–7.42) and medial meniscal extrusion (OR: 2.97, 95% CI: 2.79–3.15) as summarized in Table 6. Additionally, also lateral tibial coverage yielded one of the highest ORs (OR: 2.64, 95% CI: 2.52–2.77). The lowest ORs were achieved for MEAS features related to medial tibial cartilage volume (OR: 1.23, 95% CI: 1.20–1.26), LM volume (OR: 1.19, 95% CI: 1.18–1.19), and MM volume (OR: 1.13, 95% CI: 1.11–1.15).

**Table 6:** TKR prediction via logistic regression using MEAS features. The odds ratios are provided for each feature.

MEAS feature	Odds ratio	95% confidence interval
*TC*_*mTC*_	7.04	6.70 to 7.42
*E*_*mM*_	2.97	2.79 to 3.15
*TC*_*lTC*_	2.64	2.52 to 2.77
*A*_*mM*_	2.16	2.05 to 2.26
*V*_*FC*_	2.09	2.02 to 2.18
*V*_*lTC*_	2.08	1.98 to 2.18
*V*_*TC*_	1.74	1.68 to 1.79
$R_{A,V}^{mM}$	1.64	1.57 to 1.71
*E*_*lM*_	1.60	1.54 to 1.66
*A*_*lM*_	1.46	1.39 to 1.50
$R_{A,V}^{lM}$	1.45	1.39 to 1.50
*V*_*mTC*_	1.23	1.20 to 1.26
*V*_*lM*_	1.19	1.18 to 1.19
*V*_*mM*_	1.13	1.11 to 1.15

## Discussion

Based on our segmentations we analyzed the potential of a set of features for FB, TB, mM, and lM as biomarkers for classification of KLG and JSN and for prediction incident KOA and TKR. Compared to the related work, our classification of KOA aspects yielded similar BA as the method of Nasser et al. [33] for a determination of early KOA (classification of KLG 0 vs. KLG 2—theirs: 82.52%, ours: 84%). However, our kappa was lower than the ones of Tiulpin et al. [34] (5-class classification KLG—theirs: 0.83, ours: 0.78) and Ngyuen et al. [35] (5-class classification KLG—theirs: 0.88, ours: 0.78). For prediction of TKR, a slightly higher AUC was achieved than Eckstein et al. [36] (prediction of TKR—theirs: 0.66, ours: 0.74), but a lower AUC than Tolpadi et al. [37] (prediction of TKR—theirs: 0.83, ours: 0.74). Judging the results achieved by the features investigated in this study, one should consider that (i) the features were computed solely based on our segmentations, i.e., without taking any gray-value intensity information into account, and (ii) the features were computed from MRI data which are (in contrast to X-Ray) not acquired with the knee being in a load-bearing condition. The evaluation of KLG and JSN from X-Rays is highly depending on the rater [38] which can result in kappa statistics between 0.56 and 0.67 [39, 40]. MRI offers additional potential to investigate 3-D features, e.g. of the anatomies’ shapes, and should thus be preferred for investigation of novel biomarkers.

Considering the potential of shape encodings of the individual anatomies as biomarkers, the tibial bone showed in tendency the lowest descriptiveness for all analyzed aspects of KOA. This confirms the findings of Neogi et al. [41] who also found that shape variations of the tibial bone are either hard to capture with a low-dimensional encoding or are not as prominent and clear as features of the femoral bone. The representation of the femoral bone showed best results among all individual anatomies for differentiation of KLG. LDSE-mM performed best among the individual structures’ representations for a classification of medial JSN and LDSE-lM for a classification of lateral JSN confirming the importance of the menisci in the context of JSN [42]. In summary, the combined encoding LDSE-COMB consisting of features of femoral bone, tibial bone and meniscus yielded better results than any individual encoding of the structures of interest. This highlights the need for a holistic assessment of the entire knee. In future, further analysis of the shape of bones and menisci should be performed, i.e., analyzing the shape of sub-groups of the population in comparison to the mean shape [10]. Such an analysis may form the basis to detect clusters of similar shapes and to identify patterns within the progression of the disease [43].

In all cases (except the classification of lateral JSN) we observed that the results of LDSE-COMB can be further improved by adding MEAS features. Especially the position of the menisci can not be directly explained by a shape encoding and thus the importance of this feature is confirmed. For prediction of TKR, the potential of the MEAS features was further shown. The position of the menisci reflected in the tibial coverage and meniscal extrusion yielded the highest ORs. Volume measurements were less important with respect to TKR prediction.

As far as it concerns the detection of patterns within medical image data, we have great conviction that deep learning is the most promising approach. However, CNNs are relatively agnostic to shape information and focus mainly on the texture of images [44]. Moreover, the decision process of CNNs can be influenced by subtle changes in image information which can be hardly noticed by humans [45]. Thus, the application of deep learning for diagnostics in clinical practice remains a challenge, since results need to be explainable [46]. Furthermore, to demonstrate generalizability, deep-learning based algorithms have to be evaluated in prospective studies using large datasets acquired at different institutions with different imaging parameters and different imaging hardware [11]. Deep learning frameworks often already fuse image data with meta information using concepts e.g. of input level information fusion to add additional knowledge and improve the classification accuracy [37, 47, 48]. The features we investigated could be merged into deep learning frameworks similar as in [37, 47, 48] to provide additional insights wrt. the shape of an anatomical structure. Moreover, in contrast to pure machine learning-based approaches, the features we evaluated in this study are explainable: The semantic segmentations which are the basis of our features can be inspected in 3-D. Additionally, the measurements MEAS can be visualized in the image data and the mean shapes required for LDSE features as well as the modes of variation can be visually explored [28].

With this publication we will make our segmentations publicly available (https://pubdata.zib.de). By using our results, developments in image-based KOA biomarkers for different structures of the knee can be fostered. Our segmentation masks have been created in a fully automated manner. Quality assurance has been employed in order to omit suspicious segmentations. However, there might be still some potential for improvement e.g. in regions of high morphological variability or inhomogeneous image appearance which could be corrected manually or by novel, automated methods. Other researchers are invited to use the provided segmentations as well as to report errors or correct them and transfer the corrected data back in order to update the collection of segmentations. We envisage a platform that enables other researchers to utilize all data contained in the OAI database and to make it easily accessible for future research.

## Supporting information

S1 Fig Flow chart of quality assurance and of the data selection process for data inclusion. (PDF)

S1 Table Number of shapes which are utilized for the computation of LDSE features. Our method for automated segmentation yielded triangulated meshes for 46,996 MRI datasets contained in the OAI database. Each time point of the OAI database is analyzed independently to avoid inter-subject correlations between the shapes.
(PDF)

S2 TableSummary of MEAS and LDSE features.MEAS contains computations of the volume (*V*_*i*_), surface area (*A*_*i*_), the ratio of volume and surface area ($R_{A,V}^{i}$), meniscal extrusion *E*_*i*_, and tibial coverage *TC*_*i*_ for the respective anatomy *i*. LDSE contains features encoding the geometry of the distal femoral bone (LDSE-FB), proximal tibial bone (LDSE-TB), medial meniscus (LDSE-mM), lateral meniscus (LDSE-lM), or a combined representation of all 4 anatomies (LDSE-COMB).(PDF)

S3 Table Classification of KLG: v00-v24. (PDF)

S4 Table Classification of KLG: v36-v72. (PDF)

S5 Table Classification of KLG: v96. (PDF)

S6 Table Classification of JSN: v00-v36. (PDF)

S7 Table Classification of JSN: v48-v96. (PDF)
