# Supplementary material for: Towards novel osteoarthritis biomarkers: Multi-criteria evaluation of 46,996 segmented knee MRI data from the Osteoarthritis Initiative
Source: PLoS One. 2021 Oct 21;16(10):e0258855. doi: 10.1371/journal.pone.0258855 (PMC8530341; doi:10.1371/journal.pone.0258855)
Supplement: S1 Fig — (PDF) [file pone.0258855.s001.pdf]

*S1 Figure: Flow chart of quality assurance and of the data selection process for data inclusion.*

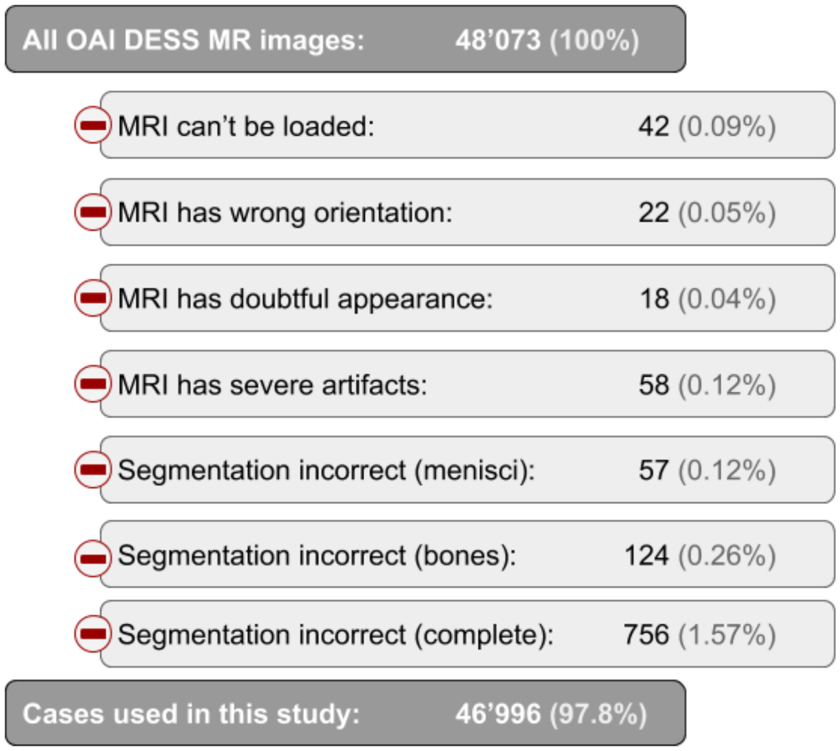


Figure 1: Flow chart of quality assurance and data selection process for data inclusion.
