# Supplementary material for: Towards novel osteoarthritis biomarkers: Multi-criteria evaluation of 46,996 segmented knee MRI data from the Osteoarthritis Initiative
Source: PLoS One. 2021 Oct 21;16(10):e0258855. doi: 10.1371/journal.pone.0258855 (PMC8530341; doi:10.1371/journal.pone.0258855)
Supplement: S1 Table — Our method for automated segmentation yielded triangulated meshes for 46,996 MRI datasets contained in the OAI database. Each time point of the OAI database is analyzed independently to avoid inter-subject correlations between the shapes. (PDF) [file pone.0258855.s002.pdf]

S1 Table: Number of shapes which are utilized for the computation of LDSE features.

Table S1: Number of shapes which are utilized for the computation of LDSE features. Our method for automated segmentation yielded triangulated meshes for 46,996 MRI datasets contained in the OAI database. Each time point of the OAI database is analyzed independently to avoid inter-subject correlations between the shapes.

**OAI time point**	v00	v12	v24	v36	v48	v72	v96
**Number of shapes**	9,345	8,025	7,338	5,500	6,616	5,413	4,759
