# Supplementary material for: Towards novel osteoarthritis biomarkers: Multi-criteria evaluation of 46,996 segmented knee MRI data from the Osteoarthritis Initiative
Source: PLoS One. 2021 Oct 21;16(10):e0258855. doi: 10.1371/journal.pone.0258855 (PMC8530341; doi:10.1371/journal.pone.0258855)
Supplement: S2 Table — MEAS contains computations of the volume (Vi), surface area (Ai), the ratio of volume and surface area (RA,Vi), meniscal extrusion Ei, and tibial coverage TCi for the respective anatomy i. LDSE contains features encoding the geometry of the distal femoral bone (LDSE-FB), proximal tibial bone (LDSE-TB), medial meniscus (LDSE-mM), lateral meniscus (LDSE-lM), or a combined representation of all 4 anatomies (LDSE-COMB). (PDF) [file pone.0258855.s003.pdf]

Table S2: Summary of MEAS and LDSE features. MEAS contains computations of the volume (V_i), surface area (A_i), the ratio of volume and surface area ($R^i_{A,V}$), meniscal extrusion E_i, and tibial coverage TC_i for the respective anatomy i. LDSE contains features encoding the geometry of the distal femoral bone (LDSE-FB), proximal tibial bone (LDSE-TB), medial meniscus (LDSE-mM), lateral meniscus (LDSE-lM), or a combined representation of all 4 anatomies (LDSE-COMB).

**Measurements MEAS (based on segmented voxels and surfaces)**
Volume: V_{FC}, V_{mTC}, V_{lTC}, V_{TC}, V_{mM}, V_{lM}
Area: A_{mM}, A_{lM}
Ratio: $R^{mM}_{A,V}$, $R^{lM}_{A,V}$
Extrusion: E_{mM}, E_{lM}
Coverage: TC_{mTC}, TC_{lTC}

**Shape encoding LDSE (based on surfaces)**
LDSE-FB (300 features)
LDSE-TB (300 features)
LDSE-mM (300 features)
LDSE-lM (300 features)
LDSE-COMB ($4 \times 75 = 300$ features)
