# Supplementary material for: Towards novel osteoarthritis biomarkers: Multi-criteria evaluation of 46,996 segmented knee MRI data from the Osteoarthritis Initiative
Source: PLoS One. 2021 Oct 21;16(10):e0258855. doi: 10.1371/journal.pone.0258855 (PMC8530341; doi:10.1371/journal.pone.0258855)
Supplement: S4 Table — (PDF) [file pone.0258855.s005.pdf]

S4 Table: Classification of KLG v36-v72

Table S4: Classification of KLG: v36-v72

		**5-class**	**3-class**	**binary classification**		
TP	Features	0 vs 1 vs 2 vs 3 vs 4	[0;1] vs 2 vs [3;4]	[0;1] vs [2;3;4]	0 vs 2	0 vs 4
v36		N = 5,407	N = 5,407	N = 5,407	N = 3,402	N = 2,233
	MEAS	0.43 ± 0.04	0.60 ± 0.02	0.72 ± 0.02	0.67 ± 0.03	0.94 ± 0.03
	LDSE-FB	0.41 ± 0.04	0.65 ± 0.02	0.81 ± 0.02	0.79 ± 0.02	0.96 ± 0.02
	LDSE-FB + MEAS	0.44 ± 0.04	0.69 ± 0.03	0.83 ± 0.02	0.80 ± 0.02	0.97 ± 0.02
	LDSE-TB	0.40 ± 0.04	0.62 ± 0.03	0.80 ± 0.02	0.77 ± 0.02	0.95 ± 0.03
	LDSE-TB + MEAS	0.43 ± 0.04	0.67 ± 0.03	0.81 ± 0.02	0.78 ± 0.03	0.97 ± 0.02
	LDSE-mM	0.41 ± 0.04	0.63 ± 0.02	0.77 ± 0.02	0.75 ± 0.03	0.90 ± 0.04
	LDSE-mM + MEAS	0.46 ± 0.04	0.67 ± 0.02	0.79 ± 0.02	0.76 ± 0.02	0.97 ± 0.02
	LDSE-lM	0.36 ± 0.04	0.56 ± 0.03	0.73 ± 0.02	0.70 ± 0.03	0.87 ± 0.05
	LDSE-lM + MEAS	0.43 ± 0.05	0.65 ± 0.02	0.77 ± 0.02	0.73 ± 0.03	0.95 ± 0.03
	LDSE-COMB	0.50 ± 0.04	0.72 ± 0.02	0.84 ± 0.02	0.84 ± 0.02	0.99 ± 0.01
	LDSE-COMB + MEAS	0.51 ± 0.04	0.73 ± 0.02	0.84 ± 0.02	0.83 ± 0.02	0.99 ± 0.01
v48		N = 6,375	N = 6,375	N = 6,375	N = 4,047	N = 2,689
	MEAS	0.44 ± 0.03	0.60 ± 0.02	0.71 ± 0.02	0.67 ± 0.03	0.94 ± 0.03
	LDSE-FB	0.41 ± 0.04	0.65 ± 0.02	0.82 ± 0.02	0.79 ± 0.02	0.95 ± 0.03
	LDSE-FB + MEAS	0.45 ± 0.03	0.68 ± 0.02	0.83 ± 0.02	0.81 ± 0.02	0.98 ± 0.02
	LDSE-TB	0.41 ± 0.03	0.63 ± 0.02	0.80 ± 0.01	0.78 ± 0.02	0.93 ± 0.03
	LDSE-TB + MEAS	0.44 ± 0.04	0.67 ± 0.02	0.81 ± 0.02	0.79 ± 0.02	0.96 ± 0.02
	LDSE-mM	0.43 ± 0.03	0.63 ± 0.03	0.77 ± 0.02	0.76 ± 0.02	0.92 ± 0.03
	LDSE-mM + MEAS	0.48 ± 0.03	0.67 ± 0.02	0.78 ± 0.02	0.75 ± 0.02	0.98 ± 0.01
	LDSE-lM	0.37 ± 0.03	0.56 ± 0.02	0.73 ± 0.02	0.69 ± 0.02	0.88 ± 0.04
	LDSE-lM + MEAS	0.45 ± 0.04	0.64 ± 0.02	0.77 ± 0.02	0.72 ± 0.03	0.96 ± 0.03
	LDSE-COMB	0.51 ± 0.04	0.73 ± 0.02	0.85 ± 0.02	0.85 ± 0.02	0.99 ± 0.01
	LDSE-COMB + MEAS	0.51 ± 0.03	0.73 ± 0.02	0.85 ± 0.01	0.85 ± 0.02	0.99 ± 0.01
v72		N = 3,198	N = 3,198	N = 3,198	N = 2,113	N = 1,715
	MEAS	0.37 ± 0.15	0.58 ± 0.05	0.70 ± 0.04	0.68 ± 0.05	0.86 ± 0.18
	LDSE-FB	0.29 ± 0.16	0.51 ± 0.06	0.73 ± 0.04	0.73 ± 0.05	0.74 ± 0.19
	LDSE-FB + MEAS	0.31 ± 0.15	0.52 ± 0.06	0.74 ± 0.04	0.75 ± 0.05	0.80 ± 0.17
	LDSE-TB	0.28 ± 0.13	0.50 ± 0.06	0.72 ± 0.03	0.69 ± 0.05	0.64 ± 0.22
	LDSE-TB + MEAS	0.30 ± 0.14	0.52 ± 0.06	0.73 ± 0.04	0.71 ± 0.05	0.76 ± 0.21
	LDSE-mM	0.31 ± 0.14	0.56 ± 0.05	0.72 ± 0.04	0.72 ± 0.05	0.69 ± 0.23
	LDSE-mM + MEAS	0.33 ± 0.15	0.59 ± 0.05	0.74 ± 0.03	0.74 ± 0.04	0.79 ± 0.19
	LDSE-lM	0.25 ± 0.13	0.48 ± 0.07	0.65 ± 0.05	0.67 ± 0.05	0.68 ± 0.23
	LDSE-lM + MEAS	0.31 ± 0.15	0.57 ± 0.06	0.72 ± 0.03	0.71 ± 0.04	0.78 ± 0.19
	LDSE-COMB	0.37 ± 0.14	0.60 ± 0.06	0.79 ± 0.03	0.80 ± 0.04	0.87 ± 0.15
	LDSE-COMB + MEAS	0.38 ± 0.17	0.61 ± 0.06	0.80 ± 0.04	0.80 ± 0.05	0.90 ± 0.16
