# Supplementary material for: Towards novel osteoarthritis biomarkers: Multi-criteria evaluation of 46,996 segmented knee MRI data from the Osteoarthritis Initiative
Source: PLoS One. 2021 Oct 21;16(10):e0258855. doi: 10.1371/journal.pone.0258855 (PMC8530341; doi:10.1371/journal.pone.0258855)
Supplement: S5 Table — (PDF) [file pone.0258855.s006.pdf]

S5 Table: Classification of KLG v96

Table S5: Classification of KLG: v96

		**5-class**	**3-class**	**binary classification**		
TP	Features	0 vs 1 vs 2 vs 3 vs 4	[0;1] vs 2 vs [3;4]	[0;1] vs [2;3;4]	0 vs 2	0 vs 4
v96		N = 3,055	N = 3,055	N = 3,055	N = 2,002	N = 1,633
	MEAS	0.44 ± 0.11	0.60 ± 0.05	0.73 ± 0.04	0.68 ± 0.05	0.92 ± 0.10
	LDSE-FB	0.30 ± 0.10	0.55 ± 0.06	0.75 ± 0.04	0.72 ± 0.05	0.72 ± 0.14
	LDSE-FB + MEAS	0.32 ± 0.10	0.57 ± 0.05	0.77 ± 0.04	0.74 ± 0.05	0.78 ± 0.13
	LDSE-TB	0.26 ± 0.09	0.51 ± 0.05	0.72 ± 0.03	0.68 ± 0.05	0.62 ± 0.16
	LDSE-TB + MEAS	0.32 ± 0.11	0.55 ± 0.04	0.75 ± 0.04	0.71 ± 0.05	0.76 ± 0.14
	LDSE-mM	0.32 ± 0.09	0.56 ± 0.05	0.73 ± 0.04	0.69 ± 0.06	0.75 ± 0.14
	LDSE-mM + MEAS	0.36 ± 0.11	0.58 ± 0.05	0.76 ± 0.03	0.72 ± 0.05	0.88 ± 0.12
	LDSE-lM	0.30 ± 0.09	0.50 ± 0.06	0.68 ± 0.04	0.66 ± 0.05	0.76 ± 0.14
	LDSE-lM + MEAS	0.35 ± 0.10	0.58 ± 0.05	0.73 ± 0.04	0.69 ± 0.05	0.86 ± 0.12
	LDSE-COMB	0.37 ± 0.12	0.62 ± 0.05	0.80 ± 0.04	0.78 ± 0.05	0.91 ± 0.09
	LDSE-COMB + MEAS	0.39 ± 0.10	0.63 ± 0.06	0.81 ± 0.04	0.78 ± 0.04	0.93 ± 0.08
