# Supplementary material for: Towards novel osteoarthritis biomarkers: Multi-criteria evaluation of 46,996 segmented knee MRI data from the Osteoarthritis Initiative
Source: PLoS One. 2021 Oct 21;16(10):e0258855. doi: 10.1371/journal.pone.0258855 (PMC8530341; doi:10.1371/journal.pone.0258855)
Supplement: S6 Table — (PDF) [file pone.0258855.s007.pdf]

S6 Table: Classification of JSN: v00-v36.

Table S6: Weighted accuracy for classification of joint space narrowing for medial and lateral condyle averaged over all time points weighted by number of cases per time point - v00-v36.

Features	Joint space narrowing [0;1;2;3]							
	v00		v12		v24		v36	
	Medial	Lateral	Medial	Lateral	Medial	Lateral	Medial	Lateral
	N = 8'785	N = 8'785	N = 7'886	N = 7'886	N = 7'225	N = 7'225	N = 5'407	N = 5'407
MEAS	0.55 ± 0.05	0.60 ± 0.07	0.55 ± 0.04	0.60 ± 0.06	0.56 ± 0.05	0.60 ± 0.06	0.55 ± 0.05	0.58 ± 0.07
LDSE-FB	0.46 ± 0.05	0.43 ± 0.07	0.47 ± 0.04	0.42 ± 0.07	0.48 ± 0.05	0.42 ± 0.08	0.47 ± 0.06	0.41 ± 0.08
LDSE-FB + MEAS	0.50 ± 0.05	0.48 ± 0.08	0.52 ± 0.05	0.48 ± 0.07	0.53 ± 0.05	0.46 ± 0.07	0.52 ± 0.06	0.48 ± 0.09
LDSE-TB	0.48 ± 0.05	0.41 ± 0.08	0.47 ± 0.05	0.42 ± 0.08	0.48 ± 0.05	0.43 ± 0.07	0.46 ± 0.05	0.41 ± 0.08
LDSE-TB + MEAS	0.51 ± 0.05	0.50 ± 0.07	0.53 ± 0.05	0.50 ± 0.08	0.53 ± 0.05	0.50 ± 0.08	0.51 ± 0.06	0.48 ± 0.08
LDSE-mM	0.56 ± 0.06	0.37 ± 0.07	0.56 ± 0.05	0.35 ± 0.07	0.57 ± 0.05	0.33 ± 0.07	0.54 ± 0.05	0.32 ± 0.08
LDSE-mM + MEAS	0.59 ± 0.05	0.52 ± 0.08	0.60 ± 0.05	0.50 ± 0.07	0.60 ± 0.05	0.48 ± 0.07	0.58 ± 0.05	0.49 ± 0.08
LDSE-lM	0.41 ± 0.05	0.57 ± 0.08	0.42 ± 0.04	0.60 ± 0.07	0.42 ± 0.05	0.56 ± 0.07	0.43 ± 0.06	0.53 ± 0.08
LDSE-lM + MEAS	0.51 ± 0.05	0.58 ± 0.07	0.53 ± 0.05	0.61 ± 0.06	0.53 ± 0.05	0.59 ± 0.07	0.52 ± 0.06	0.54 ± 0.08
LDSE-COMB	0.57 ± 0.05	0.54 ± 0.08	0.59 ± 0.04	0.54 ± 0.07	0.61 ± 0.05	0.56 ± 0.07	0.60 ± 0.05	0.54 ± 0.09
LDSE-COMB + MEAS	0.58 ± 0.05	0.55 ± 0.07	0.61 ± 0.05	0.55 ± 0.07	0.61 ± 0.05	0.58 ± 0.07	0.60 ± 0.05	0.56 ± 0.07
