# Supplementary material for: Towards novel osteoarthritis biomarkers: Multi-criteria evaluation of 46,996 segmented knee MRI data from the Osteoarthritis Initiative
Source: PLoS One. 2021 Oct 21;16(10):e0258855. doi: 10.1371/journal.pone.0258855 (PMC8530341; doi:10.1371/journal.pone.0258855)
Supplement: S7 Table — (PDF) [file pone.0258855.s008.pdf]

S7 Table: Classification of JSN: v48-v96.

Table S7: Weighted accuracy for classification of joint space narrowing for medial and lateral condyle averaged over all time points weighted by number of cases per time point - v48-v96.

Features	Joint space narrowing [0;1;2;3]					
	v48		v72		v96	
	Medial	Lateral	Medial	Lateral	Medial	Lateral
	N = 6'375	N = 6'375	N = 3'198	N = 3'198	N = 3'056	N = 3'056
MEAS	0.56 ± 0.04	0.58 ± 0.06	0.45 ± 0.15	0.44 ± 0.26	0.53 ± 0.13	0.56 ± 0.20
LDSE-FB	0.47 ± 0.05	0.41 ± 0.08	0.32 ± 0.14	0.32 ± 0.20	0.34 ± 0.14	0.38 ± 0.22
LDSE-FB + MEAS	0.52 ± 0.05	0.49 ± 0.07	0.40 ± 0.17	0.38 ± 0.25	0.39 ± 0.14	0.44 ± 0.22
LDSE-TB	0.48 ± 0.05	0.41 ± 0.07	0.37 ± 0.17	0.34 ± 0.24	0.34 ± 0.13	0.35 ± 0.25
LDSE-TB + MEAS	0.53 ± 0.04	0.47 ± 0.08	0.41 ± 0.17	0.35 ± 0.23	0.40 ± 0.13	0.42 ± 0.21
LDSE-mM	0.56 ± 0.04	0.35 ± 0.07	0.39 ± 0.17	0.34 ± 0.21	0.48 ± 0.14	0.25 ± 0.22
LDSE-mM + MEAS	0.59 ± 0.05	0.48 ± 0.07	0.44 ± 0.17	0.38 ± 0.22	0.51 ± 0.12	0.34 ± 0.22
LDSE-lM	0.43 ± 0.05	0.55 ± 0.06	0.30 ± 0.17	0.31 ± 0.19	0.37 ± 0.14	0.33 ± 0.18
LDSE-lM + MEAS	0.53 ± 0.05	0.57 ± 0.08	0.36 ± 0.18	0.32 ± 0.23	0.41 ± 0.14	0.34 ± 0.18
LDSE-COMB	0.60 ± 0.05	0.55 ± 0.07	0.47 ± 0.19	0.40 ± 0.24	0.47 ± 0.14	0.39 ± 0.22
LDSE-COMB + MEAS	0.60 ± 0.05	0.57 ± 0.07	0.45 ± 0.19	0.38 ± 0.24	0.52 ± 0.16	0.46 ± 0.23
